# TSPAN9 and EMILIN1 synergistically inhibit the migration and invasion of gastric cancer cells by increasing TSPAN9 expression

**DOI:** 10.1186/s12885-019-5810-2

**Published:** 2019-06-26

**Authors:** Yaoyue Qi, Jing Lv, Shihai Liu, Libin Sun, Yixuan Wang, Hui Li, Weiwei Qi, Wensheng Qiu

**Affiliations:** 1 Qingdao University, Qingdao, Shandong China; 2 Department of Oncology, Affiliated Hospital of Qingdao University, Qingdao, Shandong China; 3 Central Laboratory, Affiliated Hospital of Qingdao University, Qingdao, Shandong China

**Keywords:** TSPAN9, EMILIN1, Migration, Invasion, Gastric cancer

## Abstract

**Background:**

Globally, the incidence and mortality rates of gastric cancer are high, and its poor prognosis is closely related to tumor recurrence and metastasis. Therefore, the molecular mechanisms associated with the migration and invasion of gastric cancer cells are important for gastric cancer treatment. Previously, TSPAN9 has been reported to inhibit gastric cancer cell migration; however, the underlying molecular mechanism remains unclear.

**Methods:**

Human gastric adenocarcinoma cell lines, SGC7901 and AGS, were cultured in vitro. TSPAN9 expression was determined by RT-PCR, western blot analysis, and immunohistochemistry in gastric cancer and tumor-adjacent tissues. Following the over-expression and knockdown of *TSPAN9*, wound healing and cell invasion assays were performed and EMT-related protein expression was evaluated to analyze the invasion and migration of gastric cancer cells. TSPAN9 expression and the invasion and metastasis of gastric cancer cells were observed by the functional assays following *EMILIN1* over-expression.

**Results:**

Inhibiting TSPAN9 expression significantly promoted the migration and invasion of gastric cancer cells. In addition, immunofluorescence co-localization and co-immunoprecipitation analysis revealed closely related expression of EMILIN1 and TSPAN9. Moreover, EMILIN1 can synergistically boost the tumor suppressive effect of TSPAN9, which may be produced by promoting TSPAN9 expression.

**Conclusions:**

We have demonstrated that EMILIN1 induces anti-tumor effects by up-regulating TSPAN9 expression in gastric cancer. Hence, membrane proteins TSPAN9 and EMILIN1 may represent novel therapeutic targets for the treatment of gastric cancer.

**Electronic supplementary material:**

The online version of this article (10.1186/s12885-019-5810-2) contains supplementary material, which is available to authorized users.

## Background

Gastric cancer is among the most frequently encountered digestive tract tumors, and remains the fifth most common tumor in the world, as well as the second most important cause of cancer-related death [1]. Gastric cancer incidence in East Asia is significantly higher than in Western countries [2]. Although surgery can cure early disease, most patients are already in advanced stages of cancer at the time of diagnosis [3]. It is thus important that the molecular mechanisms governing gastric cancer’s development and progression be elucidated in order to better facilitate active early screening.

Tetraspanins are an evolutionarily conserved four transmembrane protein superfamily with four transmembrane segments, a small extracellular region, and a large extracellular loop (LEL) [4]. To date, 33 TSPAN proteins have been found in humans, including CD9, CD63, CD151, TSPAN1 and TSPAN8 [5]. These proteins have been shown to be involved in cell development, differentiation, movement, as well as in tumor proliferation and invasion [6, 7]. TSPAN proteins have two primary modes of interaction by which they form complexes – homologous interactions between two TSPAN molecules, and heterogeneous interactions between TSPAN and non-TSPAN proteins such as integrins or other signaling molecules [4]. The combination of TSPAN and β1 integrin is important for mediating tumor cell proliferation and invasion [5]. Most TSPAN proteins are down-regulated in metastatic tumors [5], but, conversely, some of these proteins are also elevated in the more advanced stages of cancer, such as TSPAN8, which is increased in late stages of liver cancer, gastric cancer, and rectal cancer [8]. TSPAN9, a member of the TSPAN family, is approximately 27 kD in size and has a four-transmembrane domain and has been shown to be involved in platelet aggregation and viral infection [9, 10]. Previous studies have rarely assessed its role in tumors. According to our previous studies, TSPAN9 regulates the protein secretion levels associated with tumor metastasis, such as matrix metalloproteinase-9(MMP-9) through the ERK1/2 pathway, thereby inhibiting gastric cancer cell proliferation, migration, and invasion [11].

EMILIN (also known as the Elastic Microfibril Interface Located ProteIN) is an extracellular secretory protein, with at least 4 family members (EMILIN1–4, [12]). It is highly expressed in blood vessels, lymphatic vessels and connective tissues of various organs [13]. EMILIN1 has a specifically aligned domain, including a C-terminal gc1q-like globular domain, a latent coiled-a-helical structure, and a N-terminal cysteine-rich domain [14]. Studies have shown that the gc1q homology domain of EMILIN1 can interact with α4β1 integrin, thereby affecting cell adhesion and migration [14]. What if any role is played by EMILIN1 in cancer remains uncertain. It has been found to be less expressed in breast cancer than in normal tissues, and it can inhibit the proliferation and metastasis of lung cancer cells [15, 16]. However, opposite results were obtained in ovarian serous tumors and osteosarcoma [17, 18]. To date there has been no experimental report regarding the role of EMILIN1 in gastric cancer, but it has been found to promote tumorigenesis in gastric cancer by constructing a weighted gene co-expression network [19].

We found that TSPAN9 can disrupt gastric cancer cell invasion and migration, and EMILIN1 can synergistically promote the anti-cancer effects of TSPAN9. Therefore, in assessing the anti-cancer effects of TSPAN9 and the downstream mechanism, this study found that TSPAN9 disrupts gastric cancer cell invasion and migration by inhibiting the FAK-RAS-ERK1/2 pathway by over-expressing and knocking down TSPAN9 in gastric cancer cells in vitro. EMILIN1 synergistically regulates gastric cancer cell invasion and metastasis by promoting TSPAN9 expression.

## Methods

### Cell culture

SGC7901 (Cat No. SGC-7901) and AGS (Cat No. AGS) GC cell lines came from the Shanghai Institutes for Biological Sciences (Shanghai, People’s Republic of China) and were grown in RPMI-1640 (Gibco, CA, USA) containing 10% fetal bovine serum (FBS; Thermo Fisher Scientific, MA, USA) and 1% penicillin–streptomycin (HyClone, UT, USA) under standard growth conditions. All the cell lines were kept within 10 passages and preserved in liquid N2 after receipt, and were authenticated using short tandem repeat profiling and confirmed to be free of mycoplasma prior to use.

### Human samples

We collected a total of 120 paired post-operative patient samples, with both tumor and normal tissue collected from patients who underwent operation in the Affiliated Hospital of Qing Dao University. Samples were frozen at − 80 °C until use. The Institutional Ethical Board of the Affiliated Hospital of Qing Dao University approved this study, with all patients providing written informed consent prior to sample collection.

### Cell transfection

Cells were initially cultured in the 6-well plate with a culture medium with 10% FBS for 24 h.Cells were washed once in PBS, then allowed to rest in serum-free media. We then combined the Polyethylenimine (PEI) with the indicated expression constructs for 15 min at room temperature. This solution was then added to cells for a 6–8 h period. And then, the medium was replaced with the fresh medium with 10% FBS for different times until analysis. After transfection for 48 h, the TSPAN9 expression was observed with an inverted fluorescent microscope. All reagents were used at recommended reagent to DNA ratio to transfect a constant amount of 1 μg DNA for comparison.

### Cell wound scrape assay

We seeded cells into 6-well plate, and then used a 200-μL pipette tip to create a wound in the surface of the confluent cell monolayer. The cells were then washed with PBS and incubated in serum-free media for 24 h. Thereafter, we captured images of the cells at different times over a 24 h period. A microscope was used to assess the width of the wound under × 100 magnification. Measurements of the length of the wound were performed at random intervals, and the data were analysed by ImageJ software. This experiment was conducted in triplicate.

### Cell invasion assay

Matrigel-coated Transwell cell culture chambers were used for assessments of invasion. Cells in the logarithmic phase were starved in serum-free 1640 medium for 24 h, after which they were digested by 0.25% EDTA-trypsin. The cell suspension was then treated with serum-free 1640, and a 200 μL volume of cells was added to the upper chamber in the Transwell insert (Corning Costar), while complete growth media containing serum was added into the lower chamber at 600 μL/well. Cells were then incubated for 24 h three times in duplicate. After 24 h, inserts were collected and methanol fixed for 20 min before drying. The chamber was then dyed by crystal violet for 20 min. Cells which remained in the upper chamber were gently removed using a wet cotton swab, and then the chamber was placed under an inverted microscope so that the remaining cells could be counted. The images were analysed by ImageJ software.

### Real-time PCR

RNA was extracted from cells via Trizol (TaKaRa). 1μg of this isolated RNA was then used to generate cDNA using 5 × primer script buffer, primer script enzymes, oligo dT primers and reverse transcriptase (TaKaRa). cDNA was generated through a reverse transcription reaction which was conducted at 37 °C for 15 min, 84 °C for 5 s and 4 °C for the appropriate amount of time. Real-time quantitative PCR (qPCR) was carried out using SYBR Green PCR master mix. Gene expression was normalized against β-actin. Each experiment was repeated three times and was performed independently in duplicate. mRNA expression was quantified by the cycle threshold (CT) method, and SPSS version 11.5 was used to calculate identify significant differences in the mRNA expression levels of various genes among different samples using the Mann-Whitney U test.

### Western blot analysis

From each sample, 20μg protein was separated on a 12% SDS gel for use in semi-dry western blotting. In order to block membranes, the membranes were treated with 5% BSA in TBST and then incubated with primary antibodies against β-actin-HPR (120,000, Sigma), TSPAN9 (1:1000,Abcam), p-ERK (11,000, Bioworld), EMILIN1 (11,000,Abcam), FAK (1: 1000, [1] CST), p-FAK (11,000, CST), RAS (11,000, KleanAB) overnight at 4 °C. Blots were then probed using appropriate HRP-conjugated secondary antibodies (120,000, Abcam) for 1 h at room temperature. Antibody staining was then visualized by chemiluminescence.

### Co-immunoprecipitation

Antibodies: TSPAN9 (11,000,Abcam), p-ERK (11,000, Bioworld), EMILIN1(1, 1000,Abcam). Initially, Pierce protein A agarose (Thermo Scientific, IL, USA) were washed in a solution containing 1% BSA and 10% SDS in PBS, followed by four washes with PBS. Beads were resuspended in PBS containing 1% BSA, to which 4 μl of the indicated antibody was added for 4 h at 4 °C. Beads were then washed 4 times in PBS to remove free antibody prior to immunoprecipitation. Following the immunoprecipitation reaction, samples were boiled for 3 min in 2x SDS loading buffer to elute proteins, and these proteins were separated on SDS-PAGE gels. After silver staining, mass spectrometry was used to identify the co-immunoprecipitated proteins.

### Immunofluorescence

After transfecting TSPAN9 and EMILIN1 in SGC7901 cells for 24 h, primary antibodies recognizing TSPAN9 and EMILIN1 (1500), were added to slides and incubated at 37 °C for 1 h. Slides were washed thrice in PBS, then blocked for 60 min with 20% BSA blocking reagent and then washed with 5 times with PBS. Slides were then incubated for 1 h at RT with a secondary fluorescent anti-goat antibody (11,000 in 5% BSA). ProLong Gold Antifade Mountant was used in order to mount the slides, and DAPI staining was used to identify cellular nuclei. After mounting, slides were stored at 4 °C prior to analysis.

### Statistical methods

Data are means ± standard deviation. Two-tailed unpaired Student’s t tests were used to assess significance unless stated otherwise. *P* < 0.05 was deemed significant.

## Results

### Gastric cancer cell TSPAN9 expression

**Fig. 1:**
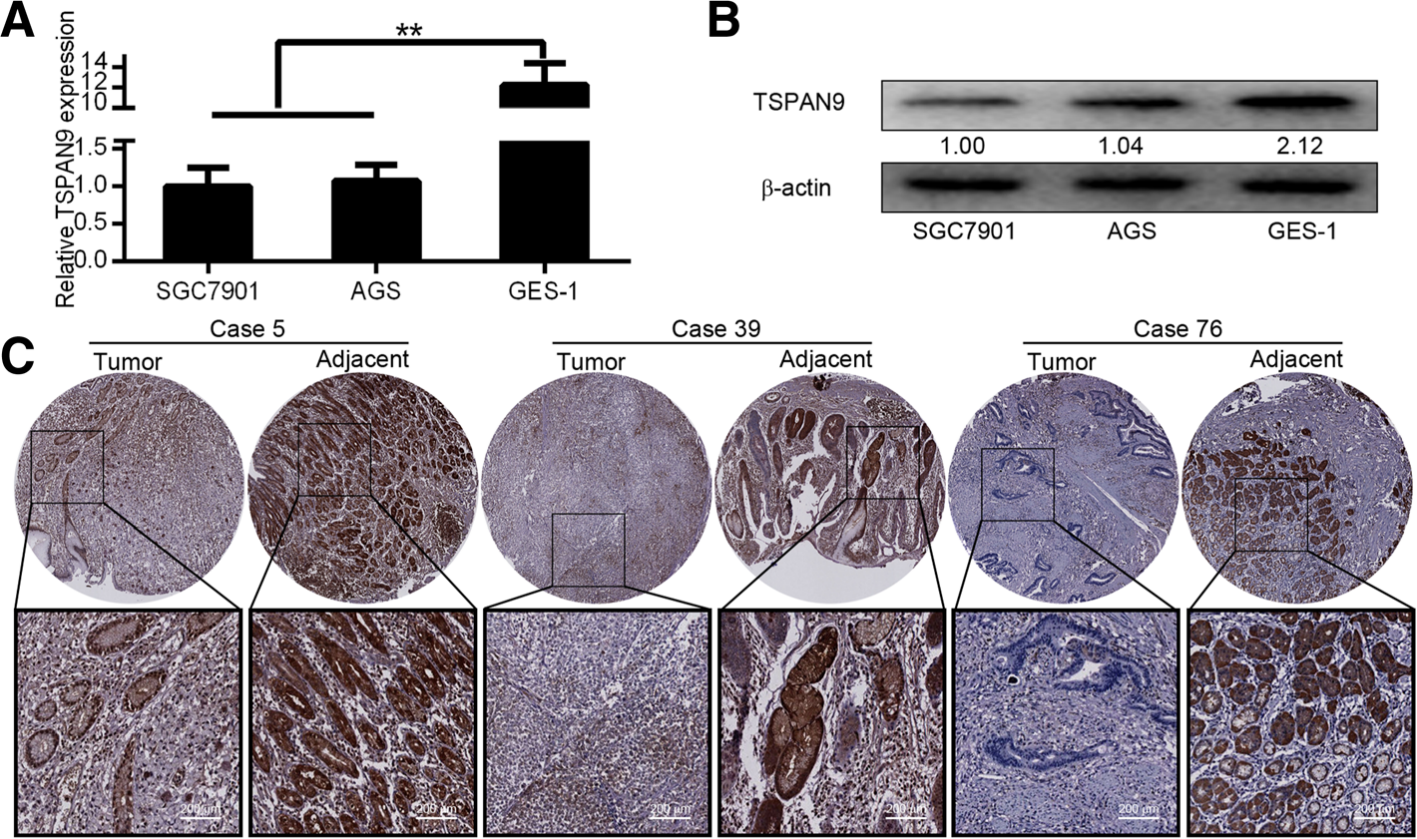
Expression of TSPAN9 in gastric cancer cells. **a** qRT-PCR was employed to assess the expression of TSPAN9 in GC cell lines (*n* = 3). GAPDH serves as an internal reference. **b** Western blot assay of TSPAN9 in GC cell lines cells, with β-actin as a loading control. **c** Immunofluorescence express the expression of TSPAN9 in GC cell lines. D. TSPAN9 staining in GC and paracancerous tissue sample. *p < 0.05, **p < 0.01, Student’s t test

Members of the TSPAN family have been shown to be overexpressed in some tumor cells [5], but currently there is no report on TSPAN9 expression in gastric cancer. Using qRT-PCR and western blotting, we therefore assessed TSPAN9 expression in gastric cancer cells, with normal human GES-1 cells as a non-cancerous control line. We observed low levels of *TSPAN9* transcripts in SGC7901 and AGS cells (Fig. 1a), and western blotting confirmed protein levels of TSPAN9 in these same cells (Fig. 1b). We then used immunohistochemistry to confirm TSPAN9 expression in gastric cancer tissue samples from patients. We observed negative TSPAN9 staining was noted in 39 of the 120 (32.5%) samples assessed from gastric cancer patients, while 81 of 120 (67.5%) samples of normal gastric tissue stained positive for TSPAN9 expression (Table 1). Representative images of TSPAN9 staining in the gastric cancer and paracancerous tissue samples are shown in Fig. 1c. Together, these results indicate that only low levels of TSPAN9 are in gastric cancer cells as well as in tissue samples.

**Table 1:**
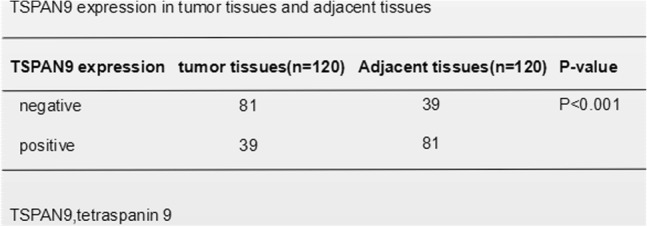
According to the manufacturer’s recommended criteria, the samples were independently scored for intensity of TSPAN9 staining by two pathologists without knowledge of the clinical outcome at × 100 and × 200 magnification

The staining intensity was divided into three grades (using a scoring system of 0–3): No staining (0), slightly yellowish (1), brownish yellow (2) and dark-brown (3). The multiplications of the two scores were graded as follows: 0 (0 score), 1+ (1–4 score), 2+ (5–8 score) and 3+ (9–12 score). Intensity scores of 0 or 1+ were designated as negative expression, whereas those of 2+ or 3+ were designated as positive expression

### TSPAN9 affects gastric cancer cell migration and invasion

**Fig. 2:**
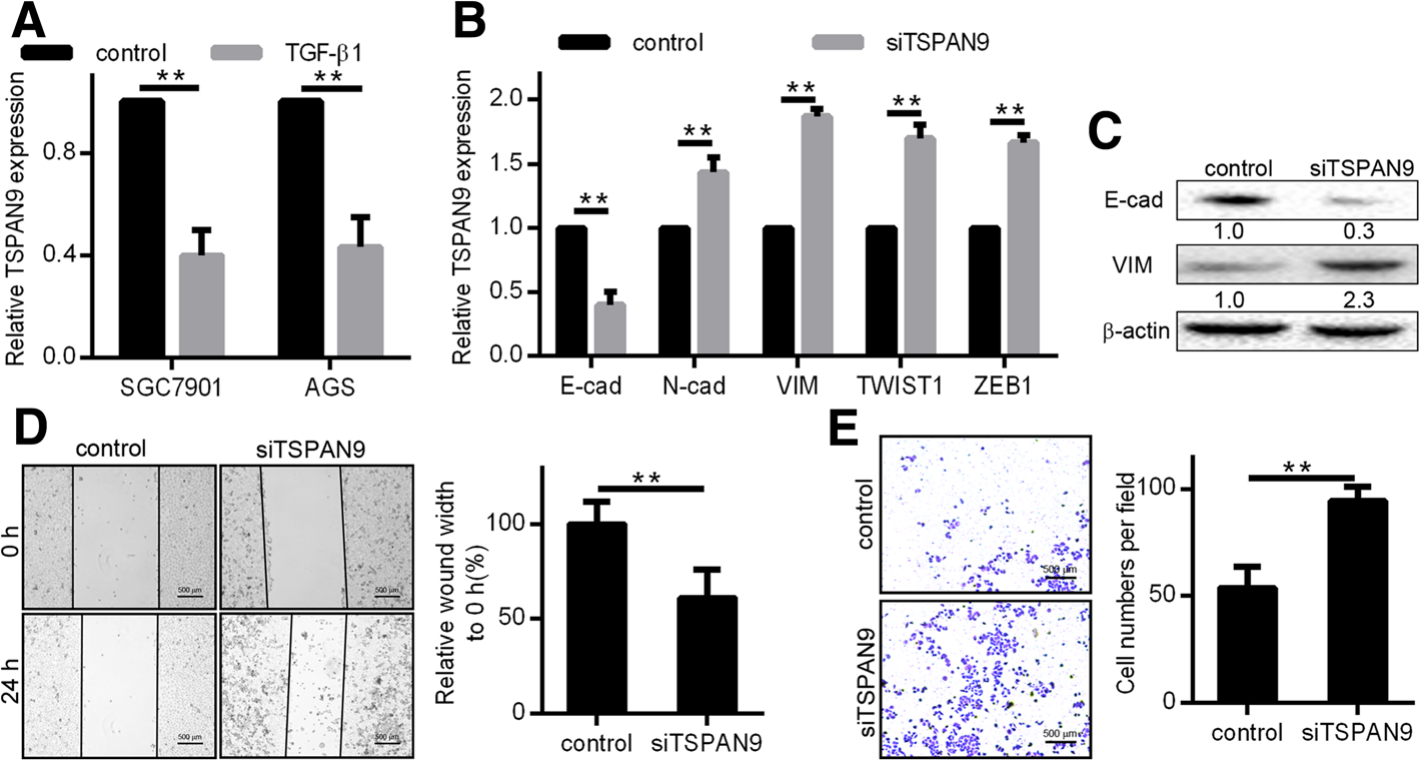
TSPAN9 affects gastric cancer migration and invasion. **a** Following a 48 h 10 ng/ml TGF-β1 treatment, TSPAN9 expression was assessed. **b** qRT-PCR was used to quantify EMT-related gene expression following *TSPAN9* siRNA transfection (or control transfection). **c** Western blotting was used to assess levels of proteins linked with the EMT following siRNA transfection. After *TSPAN9* siRNA transfection, wound-healing (**d**) and migration assays (**e**) examined how this siRNA affected GC cell migration. A total of 2 × 105 cells SGC7901 cells, si-RNA transfected subclones, and controls were used in a Transwell-based invasion assay. A total of 10 fields per insert were counted to determine the number of invading cells. *n* = 5 replicates. *p < 0.05, **p < 0.01, Student’s t test

Tetraspanins play a role in tumor cell invasion, potentially owing to the link between these proteins and integrins [20]. Integrins are vital proteins essential for allowing cells to effectively interact with the extracellular matrix (ECM), and are vital for proliferation, adhesion, migration, and differentiation of cells [21]. To assess to what extent TSPAN9 is linked with gastric cancer progression, we assessed how TSPAN9 influences the epithelial-mesenchymal transition (EMT) as well as gastric cancer cell migration. We treated SGC7901and AGS gastric cancer cells using TGF-β1, which is known to induce the EMT. TSPAN9 expression was markedly reduced in gastric cancer cells following TGF-β1 treatment (Fig. 2a). Next, we silenced TSPAN9 and found that this led to the dysregulation of multiple EMT-related markers, leading to decreased E-cadherin and increased N-Cadherin, vimentin, Twist, and ZEB1 (Fig. 2b and c). TSPAN9 further impaired the migration of SGC7901cells and AGS cells in wound healing assays (Fig. 2d). *TSPAN9* knockdown induced the migration of gastric cancer cells, consistent with the above results (Fig. 2e).

### The FAK-RAS-ERK1/2 pathway is activated via the low expression of TSPAN9

**Fig. 3:**
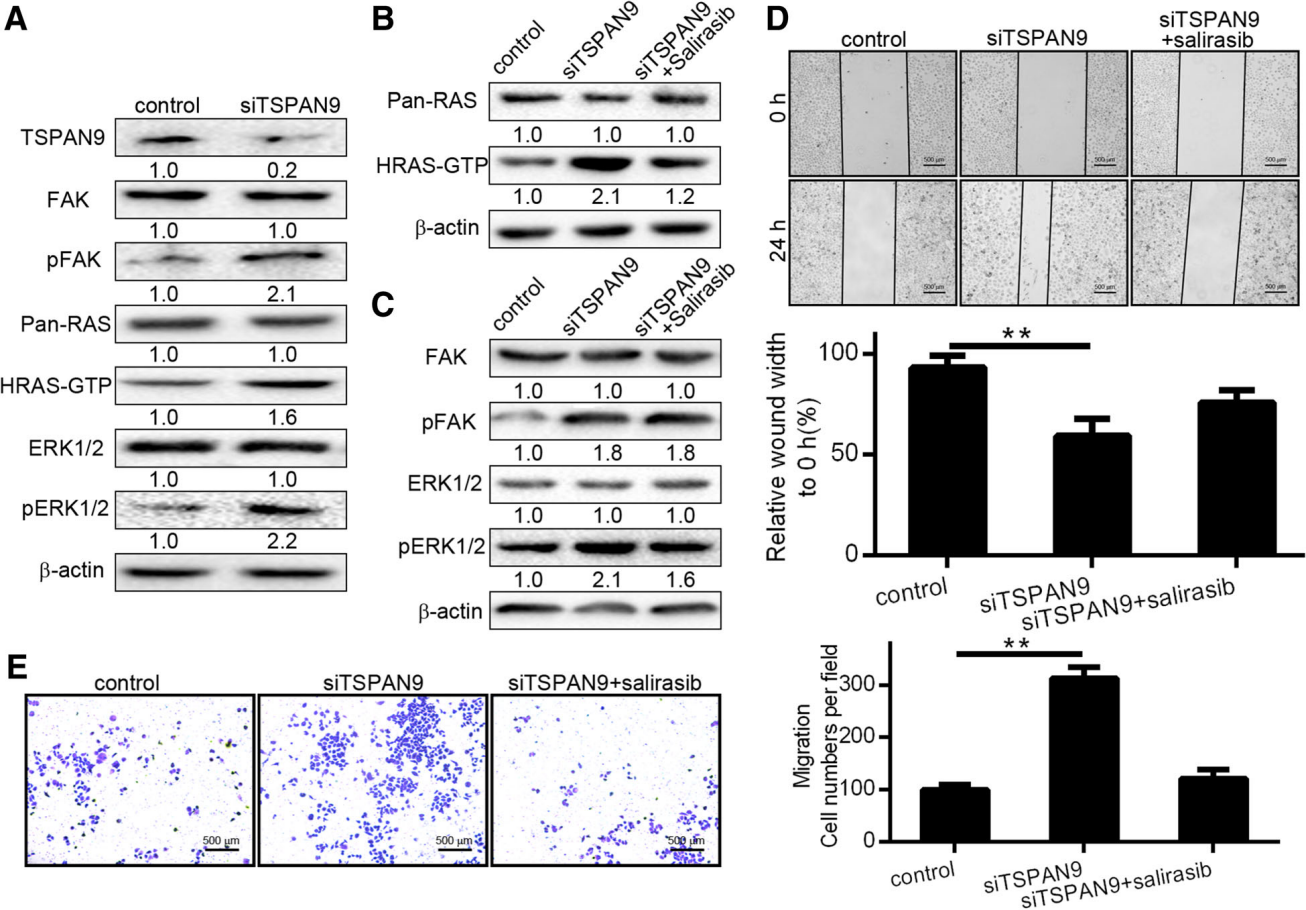
The FAK-RAS-ERK1/2 pathway is activated via the low expression of TSPAN9. **a** In SGC7901 cells transfected with a *TSPAN9* inhibitor (si-RNA) or a negative control (control) for 48 h, FAK, HRAS, and ERK1/2 levels were assessed by western blotting, with β-actin as a loading control. **b** and **c** s*i-TSPAN9* or *si-TSPAN9* and RAS inhibitor was transfected into SGC7901 cells. We them conducted wound-healing (**d**) and migration assays (**e**) to assess how *TSPAN9* siRNA and RAS inhibition affect the migration of GC cells. *p < 0.05, **p < 0.01, Student’s t test

FAK phosphorylation (Tyr925) produces an SH2-bearing molecule binding site, further triggering RAS-dependent MAP kinase pathway activation [22]. To verify the downstream effects caused by knockout of *TSPAN9*, we underexpressed TSPAN9 protein in gastric cancer cells. Compared with controls, pFAK, HRAS-GTP and pERK1/2 were significantly increased in cells with low TSPAN9 expression (Fig. 3a). Then we added the RAS inhibitor to these low TSPAN9-expressing cells. We confirmed the ability of this compound to significantly inhibit HRAS-GTP (Fig. 3b), and was found that pERK1/2 levels were decreased relative to the untreated TSPAN9 group, while the expression of pFAK was not significantly altered (Fig. 3c). To test whether these changes in invasion and migration were caused by TSPAN9-triggered cell signals, we treated TSPAN9 low-expressing cells with RAS inhibitors and observed their invasive activity. We found that RAS inhibitor treatment significantly impaired this invasive ability relative to untreated controls (Fig. 3d and e). From this we can conclude that low TSPAN9 expression can promote gastric cancer cell invasion and migration via FAK-RAS-ERK1/2 signaling.

### TSPAN9 and EMILIN1 have synergistic anti-tumor effects

**Fig. 4:**
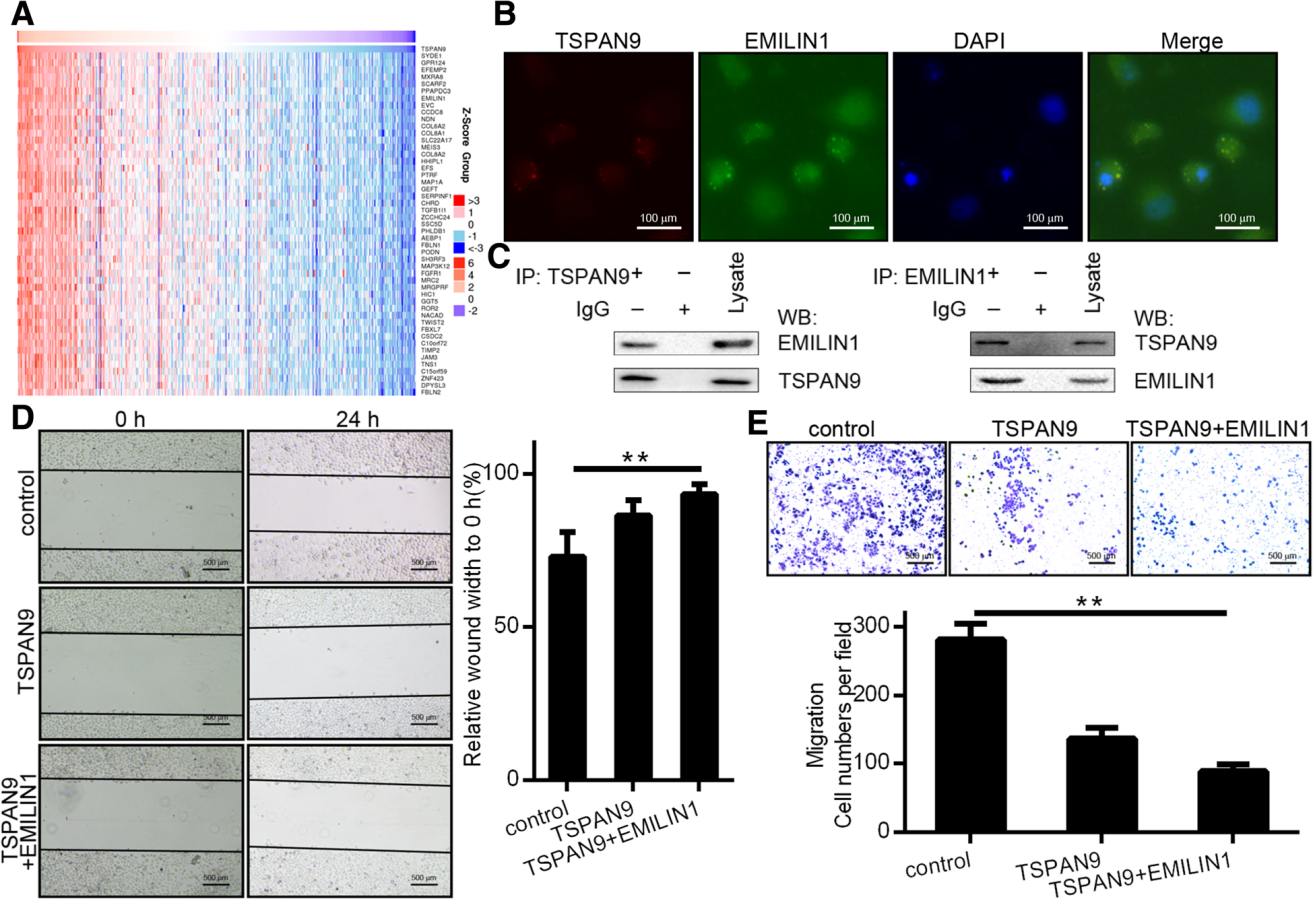
EMILIN1 and TSPAN9 have synergistic anti-tumor effects. **a** A heatplot map of gene expression levels of the top 15 upregulated genes in GC tissues. **b** *TSPAN9* and *EMILIN1* plasmids were transfected into SGC7901 cells for 24 h, and immunofluorescence was employed to establish the location of TSPAN9 and EMILIN1 in SGC7901 cells. **c** SGC7901 cells were transfected with *TSPAN9* and *EMILIN1* plasmids, and for co-immunoprecipitation of TSPAN9 complexes was then conducted in whole cell protein lysates. Immunoprecipitated products were separated via SDS-PAGE and stained using silver staining reagent. The extracted peptide was sequenced by mass spectrometry. *TSPAN9* plasmids or *TSPAN9* and *EMILIN1* plasmids were transfected into SGC7901 cells, and then wound-healing (**d**) and migration assays (**e**) were conducted to assess how TSPAN9 and TSPAN9 + EMILIN1 affect GC cell migration. *p < 0.05, **p < 0.01, Student’s t test

To explore the genes involved in TSPAN9 signaling, we analyzed gene expression profiles. We analyzed the genes that co-expressed with TSPAN9 in the TCGA database by using the online tool “Linkedomics” and generate a heatmap of *TSPAN9*-related genes. The heatmap shows a positive correlation between *EMILIN1* and *TSPAN9* expression (Fig. 4a). We then performed immunofluorescence measurements of EMILIN1 and TSPAN9 in cells and found that they co-localized in gastric cancer cell (Fig. 4b). To verify this co-localization, we assessed potential interactions between TSPAN9 and EMILIN1 by co-immunoprecipitation (co-ip). After immunoprecipitation with anti-TSPAN9 antibodies, we were able to detect EMILIN1 by western blotting, indicating that EMILIN1 and TSPAN9 are associated in protein complexes within cells (Fig. 4c). We respectively overexpressed EMILIN1 and TSPAN9, and found that overexpressing EMILIN1 exclusively had no significant effect on tumor migration and invasion (Additional file 1: Figure S1c and d), while overexpressing TSPAN9 could significantly suppress tumor. It was further found that the simultaneous high expression of TSPAN9 and EMILIN1 was more inhibitory of gastric cancer cell migration and invasion than was the overexpression of TSPAN9 alone (Fig. 4d and e). Therefore, we speculate that EMILIN1 itself does not play a major role in tumor suppression, but through synergy with TSPAN9 to exert a anti-tumor effect.

### EMILIN1 exerts synergistic anti-tumor effects by promoting the expression of TSPAN9

**Fig. 5:**
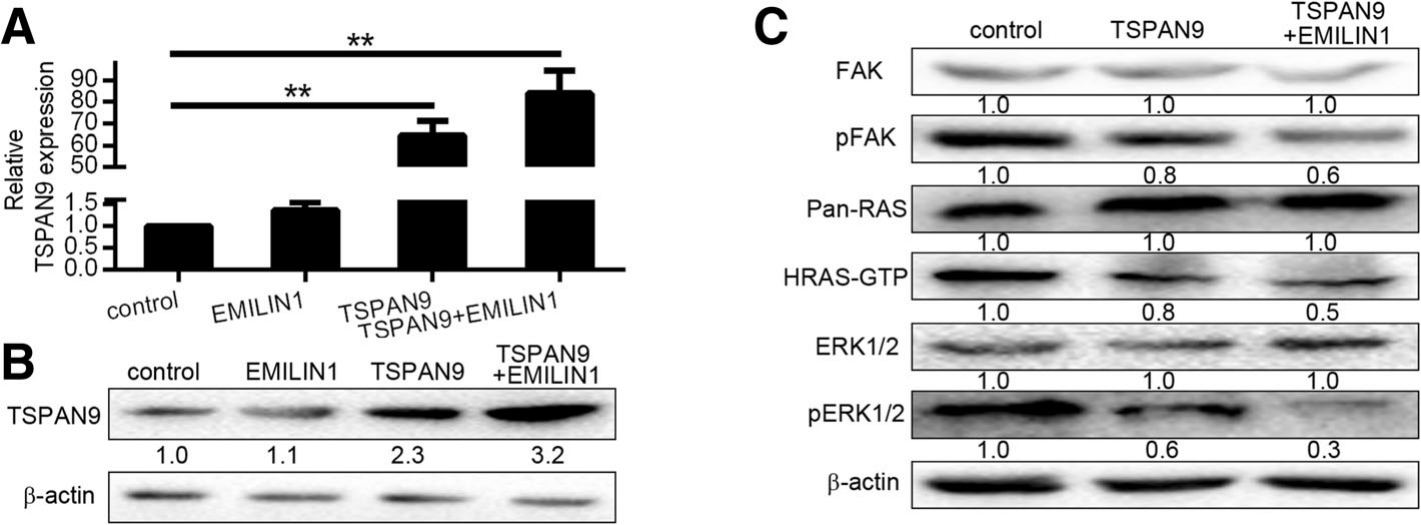
EMILIN1 exerts synergistic anti-tumor effect by promoting the expression of TSPAN9. SGC7901 cells were transfected with *TSPAN9* plasmid, *EMILIN1* plasmid, both, or with appropriate negative controls, and then qRT-PCR (**a**) and western blotting (**b**) were utilized to assess TSPAN9 expression. **c** *TSPAN9* plasmid or *TSPAN9* and *EMILIN1* plasmid was transfected into SGC7901 cells, and western blotting was then performed.*p < 0.05, **p < 0.01, Student’s t test

Previous studies have found that the gC1q domain of EMILIN1 can bind to α4β1 integrin, mediating downstream effects, and TSPAN family proteins are also known to be able to bind to β1 integrin [13, 23]. Therefore, we hypothesized that the synergy of EMILIN1 and TSPAN9 may be linked with the promotion of TSPAN9 expression. To that end, we overexpressed EMILIN1, TSPAN9, or both of these proteins in AGS cells. We subsequently observed the greatest amount of TSPAN9 was detectable in cells overexpressing EMILIN1 and TSPAN9 (Fig. 5a and b). However, after changing the expression level of EMILIN1, the effects on the mRNA and protein levels of TSPAN9 were not obvious (Additional file 1: Figure S1a and b). Therefore, we believe that the synergistic anti-tumor effect of EMILIN1 and TSPAN9 is mainly achieved by TSPAN9.We further found that simultaneous overexpression of EMILIN1 and TSPAN9 more significantly inhibited the FAK-RAS-ERK1/2 pathway as compared to the overexpression of TSPAN9 alone (Fig. 5c). All these results suggested that the synergistic anti-tumor effects of EMILIN1 and TSPAN9 are achieved by increasing the expression level of TSPAN9. To clarify the molecular mechanism of EMILIN1 and TSPAN9, we overexpressed TSPAN9 and EMILIN1 + TSPAN9 in gastric cancer cells, respectively. Quantitative analysis of collected proteins at different time points, it showed that the amount of protein of 48 h was less than 24 h in the TSPAN9 group. It indicated that TSPAN9 had degraded at 48 h after transfection. While in the TSPAN9 + EMILIN1 group, the expression of TSPAN9 at 48 h was not much changed compared with 24 h (Additional file 2: Figure S2), indicating that the combination of TSPAN9 and EMILIN1 can partially inhibit the degradation of TSPAN9.

## Discussion

Here, we demonstrate that the expression of the membrane protein TSPAN9 regulates gastric cancer cell migration and invasion by inhibiting the FAK-RAS-ERK1/2 signaling pathway. The extracellular secretory protein EMILIN1 can increase this tumor suppressive effect by promoting the expression of TSPAN9. Although EMILIN1 can promote the expression of TSPAN9, the premise of this promotion is that TSPAN9 reaches a certain expression level. Since the expression level of TSPAN9 in tumor cells is lower than that of normal cells, simply increasing the expression of EMILIN1 in tumor cells does not promote the expression of TSPAN9. Only by increasing the expression of TSPAN9 at the same time can the synergy be more significant. However, the more in-depth molecular mechanism between TSPAN9 and EMILIN1 need further study. The association of extracellular secreted proteins with transmembrane proteins is consistent in vitro conditions in which cells continuously interact with ECM components. This finding further explains the unique role of EMILIN1 and TSPAN9 in maintaining cell migration and metastasis in gastric cancer cells.

Although GC is a commonly encountered malignancy, the prognosis is often poor [24]. It is therefore important that novel therapeutic avenues be identified to treat this cancer. We observed significantly reduced TSPAN9 in human GC tissues relative to normal mucosal tissues, with this expression being negatively correlated both with the size of tumors and rates of lymph node metastases. Treatment with EMILIN1 resulted in the upregulation of TSPAN9. EMILIN1 has been shown to help inhibit cell proliferation in the epithelial system by activating RAS-ERK signaling. We have herein confirmed the role of EMILIN1 in inducing tumor migration and invasion. By regulating the expression of TSPAN9 in GC cells in vitro, it was found to modulate tumor EMT and migration. EMT plays an important role in embryonic development, tissue remodeling and wound healing, and usually occurs at the aggressive front of metastatic tumors [25]. During EMT, epithelial cells lose intercellular adhesion and acquire fibroblast-like features that increase migration and invasiveness [26]. Therefore, the analysis of EMT is necessary when studying the invasion and migration of cancer.

In advanced cases of gastric cancer, tumor invasion and metastasis are the main influencing factors leading to poor prognosis and recurrence [27]. Therefore, finding an effective target that affects these activities is an important way to treat advanced gastric cancer. We have found that TSPAN9 acts as a 4 transmembrane protein involved in the regulation of both the migration and proliferation of cells in both cancerous and normal cells. While transmembrane proteins are closely related to integrins [23], TSPAN9 has been found to inhibit EMT in tumor cells, and the role of integrins in this warrants further exploration. Pathways including Wnt/β-catenin, NOTCH1, and TGF-β-SMAD signaling can all mediate the broad transcriptional changes associated with the EMT [28]. These signaling pathways, as well as activated secondary pathways such as PI3K-AKT, RAS/MAPK, p38 MAPK and JUN N-terminal kinases, work together to regulate the proliferation, migration, and angiogenesis associated with the growth and development of tumors [29, 30]. Our experiments have found that TSPAN9 can inhibit the RAS/ERK pathway, thereby inhibiting tumor invasive and metastatic potential. The RAS/ERK pathway is linked with many activities advantageous to tumors, including those closely related to tumor autophagy and apoptosis [31, 32]. In future studies, we will therefore focus on how these proteins relate to autophagy in tumors. In this study, immunofluorescence and co-ip analysis demonstrated the close relationship between EMILIN1 and TSPAN9 in protein expression level, so we have reason to believe that the presence of TSPAN9-EMILIN1 complex and this complex may mediate downstream signaling pathways to regulate tumor migration and invasion.

Although the exact mechanisms of EMILIN1-related TSPAN9 regulation remains to be elucidated, we hypothesize that integrins mediate the interaction of cells with the ECM and further promote TSPAN9 expression. In summary, in the present study we highlighted the roles of EMILIN1 and TSPAN9 in tumor cell regulation and the mechanisms involved in TSPAN9-mediated cell migration and invasion.

## Conclusions

We have demonstrated that EMILIN1 induces anti-tumor effects by up-regulating TSPAN9 expression in gastric cancer. Therefore, we have highlighted the role of EMILIN1 and TSPAN9 in cancer progression. In addition, the EMILIN1 and TSPAN9 membrane proteins may represent novel therapeutic targets for the treatment of GC.

## Additional files

Additional file 1: **Figure S1.** A. Following a 24 h *siEMILIN1* treatment, TSPAN9 expression was assessed. B. Western blotting was used to assess levels of TSPAN9 protein following siRNA transfection. After *TSPAN9* siRNA transfection, wound-healing (C) and migration assays (D) examined how EMILIN1 affected GC cell migration. *p < 0.05, **p < 0.01, Student’s t test. (TIF 20243 kb)

Additional file 2: **Figure S2.** A. Western blotting was used to assess levels of TSPAN9 following *TSPAN9* and *EMILIN1* transfection, and samples were taken at12, 24, and 48 h time points. (TIF 4437 kb)

## Data Availability

All data generated or analysed during this study are included in this published article [and its supplementary information files].

## Abbreviations

co-ip: Co-immunoprecipitation
ECM: Extracellular matrix
EMILIN: Elastic Microfibril Interface Located ProteIN
EMT: Epithelial-mesenchymal transition
LEL: Large extracellular loop
MMP-9: Matrix metalloproteinase-9
