# Supplementary figures and images for: TSPAN9 and EMILIN1 synergistically inhibit the migration and invasion of gastric cancer cells by increasing TSPAN9 expression
Source: BMC Cancer. 2019 Jun 26;19:630. doi: 10.1186/s12885-019-5810-2 (PMC6595627; doi:10.1186/s12885-019-5810-2)

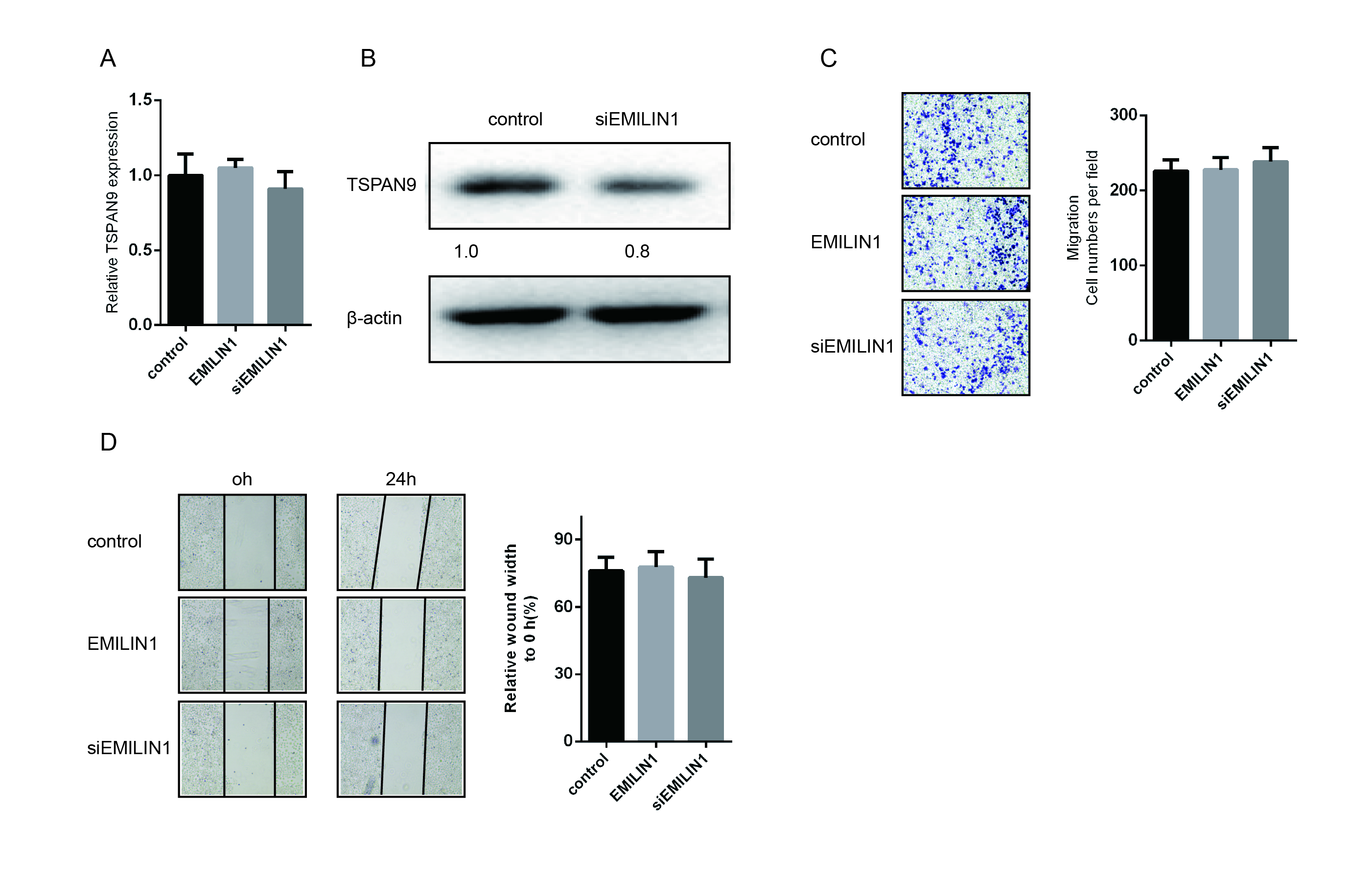

Supplement: Supplementary file 1 — Figure S1. A. Following a 24 h siEMILIN1 treatment, TSPAN9 expression was assessed. B. Western blotting was used to assess levels of TSPAN9 protein following siRNA transfection. After TSPAN9 siRNA transfection, wound-healing (C) and migration assays (D) examined how EMILIN1 affected GC cell migration. *p < 0.05, **p < 0.01, Student’s t test. (TIF 20243 kb) [file 12885_2019_5810_MOESM1_ESM.tif]

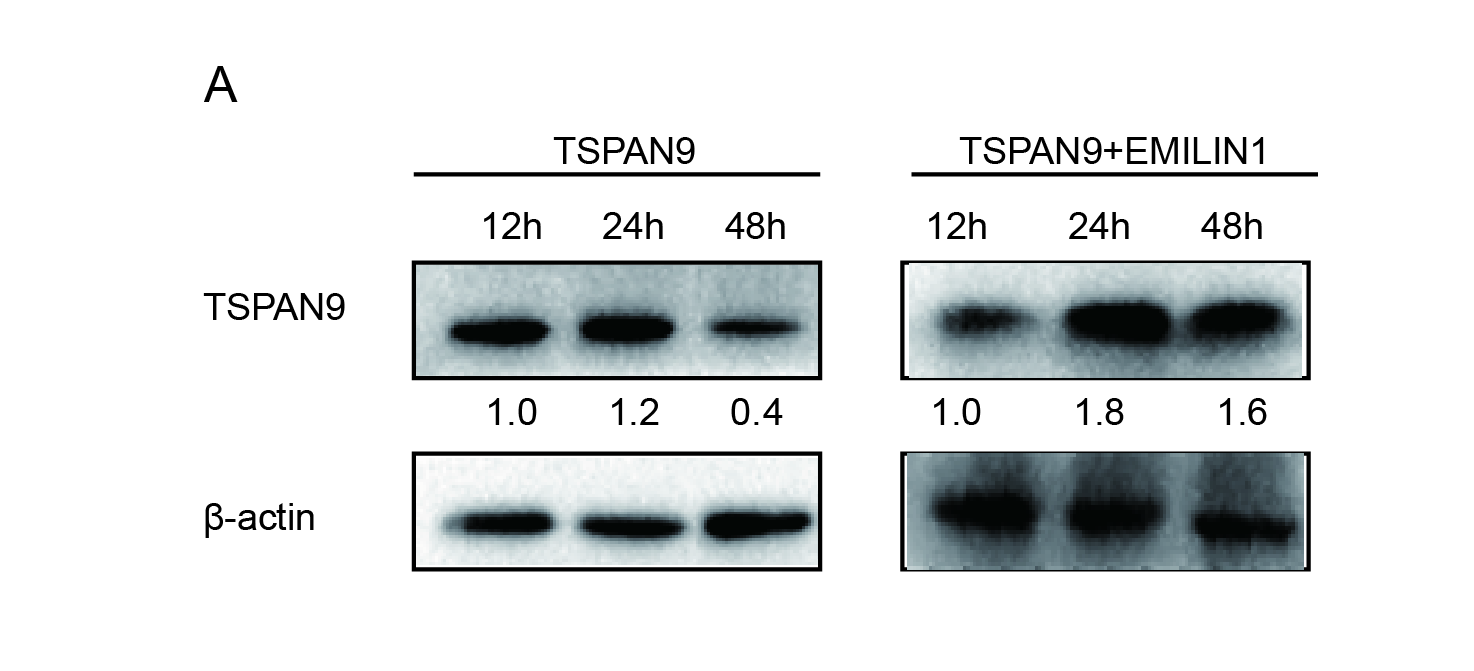

Supplement: Supplementary file 2 — Figure S2. A. Western blotting was used to assess levels of TSPAN9 following TSPAN9 and EMILIN1 transfection, and samples were taken at12, 24, and 48 h time points. (TIF 4437 kb) [file 12885_2019_5810_MOESM2_ESM.tif]
